# Novel acaricidal and growth-regulating activity of *Aloe vera* and *Rheum rhabarbarum* extracts and their oil/water nanoemulsions against the camel tick*, Hyalomma dromedarii*

**DOI:** 10.1038/s41598-023-43776-6

**Published:** 2023-10-05

**Authors:** Ibrahim T. Radwan, Randa I. Eltaly, Mohamed M. Baz, Mohamed Yousif, Abdelfattah Selim, Hanan A. A. Taie, Eman A. Manaa, Hanem F. Khater

**Affiliations:** 1 Supplementary General Sciences Department, Faculty of Oral and Dental Medicine, Future University in Egypt, Cairo, 11835 Egypt; 2 Zoology and Entomology Department, Faculty of Science, Al-Azhar University, Cairo, 11651, Egypt; 3 Department of Entomology, Faculty of Science, Benha University, Benha, 13518 Egypt; 4 Parasitology Department, Faculty of Veterinary Medicine, Benha University, Benha, 13736 Egypt; 5 Department of Animal Medicine (Infectious Diseases), Faculty of Veterinary Medicine, Benha University, Benha, 13736 Egypt; 6 Plant Biochemistry Department, Agricultural and Biology Research Institute, National Research Center, 33 El-Bohouth St. (Former El-Tahrir St.), Dokki, Cairo, 12622 Egypt; 7 Animal and Poultry Production, Department of Animal Wealth Development, Faculty of Veterinary Medicine, Benha University, P.O. Box 13736, Toukh, Qalyubia Egypt

**Keywords:** Biological techniques, Zoology, Nanoscience and technology

## Abstract

*Hyalomma dromedarii* is an important tick species infesting livestock. This work evaluated the novel adulticidal, insect growth-regulating, and enzymatic efficacy of ethanol plant extracts of *Aloe vera* and *Rheum rhabarbarum* and their nanoemulsions against males and engorged females of the camel tick, *H. dromedarii*. The physicochemical properties of nanoemulsions were evaluated. The High-Performance Liquid Chromatography (HPLC) analyses indicated that the extracts contained polyphenols and flavonoids, which could enhance their acaricidal effect. Dynamic light scattering (DLS) of the nanoemulsions of *A. vera* and *R. rhabarbarum* were 196.7 and 291 nm, whereas their zeta potentials were − 29.1 and − 53.1 mV, respectively. Transmission electron microscope (TEM) indicated that nanoemulsions showed a regular spherical shape (less than 100 nm). Fifteen days post-treatment (PT) with 25%, the mortality% of *A. vera* and *R. rhabarbarum* were 88.5 and 96.2%, respectively. Five days PT, the median lethal concentration values of *A*. *vera, R. rhabarbarum,* and their nanoemulsions were 7.8, 7.1, 2.8, and 1.02%, respectively, and their toxicity indices were 91.02, 100, 36.4, and 100%, respectively. Their median lethal time values PT with 3.5% were 6.09, 5.09, 1.75, and 1.34 days, respectively. Nanoemulsions enhanced the efficacy of the crude extract 1–7 folds, 5 days PT, and accelerated their speed of killing ticks 2–4 times. The total protein and carbohydrates, Acetylcholinesterase, Alpha esterase, and Amylase were affected PT. The reproductive potential of engorged females was adversely impacted. In conclusion, the novel *A. vera* and *R. rhabarbarum* extracts were promising acaricides, and their nanoformulations enhanced their efficacies.

## Introduction

Ticks are serious ectoparasites transmitting severe infectious diseases^1–7^. *Hyalomma dromedarii* is a blood-feeding ectoparasite affecting livestock and causes severe economic losses because of retarded growth, weight loss, decreased milk and meat production, and transmission of serious diseases^8^. Various approaches for controlling ticks are dependent mainly on applying different conventional acaricides^9^. Despite their effectiveness, the development of resistance against many commercially available acaricides has been reported for *H. dromedarii* in Egypt^10,11^.

There is a persistent demand for searching for eco-friendly control strategies using Plant-based resources (botanicals), biological control agents, vaccination, photosensitizers, and safe acids^12–18^ to reduce the health and environmental hazards resulting from repeated applications of conventional pesticides^19–22^. Botanicals have been widely used, since ancient civilizations^23^, because of their high efficacy against pests and illness^14,24–32^, relative safety to non-target organisms^27,33,34^, rapid biodegradation, and prevention of development of resistance in pests because of their various active substances and mechanisms of actions^19–21,35^. Against pests of medical and veterinary importance, botanicals induce ovicidal^36,37^, larvicidal^38–43^, adulticidal^36,44–48^, repellent^36,45,46^, and insect growth regulating (IGR) effects^38,49–51^.

Secondary metabolites are chemical components that many plants produce such as flavonoids, phenols, carbohydrates, alkaloids, glycosides, saponins glycosides, amino acids, enzymes, tannins, essential oils, and pectins^28,47,52–54^. Botanicals contain numerous active ingredients acting as bio-pesticides, specifically as nematicides, protoscolicidal, fungicides, insect development regulators, and anti-feedants with toxicity to arthropod pests^19–21,55–57^.

Aloe, *Aloe vera* (L.) Burm.f. (Asphodelaceae) is a cactus-like plant used in numerous medicinal and cosmetic products^58^. It was used by the Ancient Egyptians to treat skin problems and scabies and used as pesticides and repellents^30^. It eases gastrointestinal disorders and dermal diseases. It has antiviral, antifungal, antibacterial, anti-inflammatory, antitumor, and immunomodulatory effects. *A. vera* contains diversified active components like essential and nonessential amino acids, chromones (isoaloeresin D and Isorabaichromone); carbohydrates (acetylated mannan and glucomannan); vitamins (B1, B2, α-tocopherol, and folic acid); enzymes (alkaline phosphatase, amylase,…etc); some other organic-based substances (arachidonic acid, linolic acid, triterpenoids, and lignin); and good command of organic-based active ingredients (syringic, sinapinic acid, myricetin, acid, and vanillic acid)^53,58^.

Rhubarb, *Rheum rhabarbarum* (L.) (Polygonaceae) is a medicinal plant grown worldwide (particularly in Asia) for its substantial edible petioles. Rhubarbs have long been, since the Middle Ages, used in Asia, Europe, and other places because of their antimicrobial, anti-inflammatory, antispasmodic, antioxidant, mucolytic, purgative, sedative, cardioprotective, and blood detoxifying efficacies. Moreover, *R. rhabarbarum* is a tough perennial herbaceous plant with close-knit and dense rhizomes. It has tuberous roots, numerous branches, triangular leaves, and long stems. It contains many biologically active compounds, the most important of which are stilbenes, flavonoids, and anthraquinones^59^. Rhapontigenin extracted from *Rheum undulatum* acts as an antioxidant protecting cells from damage caused by oxidative stress^59^ and the roots of *R. emodi* induce antioxidant and anticancer properties^60^.

Nanotechnology is recently used in fields related to humans and animals, especially pharmaceutical, food, and agricultural research developing nano-delivery systems to encapsulate, protect, and deliver lots of different compounds to achieve unique and picture-perfect outcomes. Colloidal dispersion is an important delivery system because of its compatibility, small size, good command of loading capacity, and stability because of its unique physiochemical properties and bioavailability. The diversity of encapsulated drugs comes from their nature. A microemulsion or nanoemulsion consists of two phases: aqueous phase, oil phase, and surfactant and/ or cosurfactant acting as an equalizer to assist the stability of nanoparticles. Such a structure makes nanoemulsion capable of accepting both hydrophilic and hydrophobic drug platforms. The aqueous phase consists of water beside some other phospholipids to enhance solubility and stability; whereas, the oil phase is varied according to the application, like oleic acid, ethyl oleate, mineral oils, vegetable oil, or triglycerides, which promotes a different type of microcrystalline called solid lipid nanoparticles (SLN) and their generations type II nanostructured lipid carrier (NLC) in size of 100 to 500nm if the triglyceride solid was used, but if liquid or semisolid was used at room temperature, it generates microemulsion with a relatively smaller size than those got from solid^61–63^. Sometimes oleic acid is used to obtain nanoemulsions based on natural extracts, trying to optimize the benefits of the two systems and continuing the co-author's work about nanomaterial research related to the insecticidal effect of natural resources like essential oils and plant extracts^27,34^.

Nanotechnology has been applied in numerous formulations to create many new products with faster effects and a wide range of applications in several fields such as pesticides^27,33,34,41,42,64^. This work aimed to evaluate the novel adulticidal, insect growth-regulating, and enzymatic efficacy of ethanol extracts of aloe and rhubarb and their nanoemulsions against males and engorged females of the camel tick, *H. dromedarii.*

## Materials and methods

### Plant source

Two plants, *A.vera* leaves and *R. rhabarbarum* stems were obtained from a local herbalist through a botanical specialist. Plant materials were collected according to institutional, national, and international guidelines and legislation. Plants were identified, and authenticated by Dr. Therese Labib, Botanical Specialist and Consultant of Plant Taxonomy, Department of Flora and Taxonomy, Ministry of Agriculture and Director of the Orman Botanical Garden, Giza, Egypt. Voucher specimens were deposited in the National Research Center’s Herbarium (CAIRC), Department of Phytochemistry and Plant Systematics, by Dr. Mona Mohamed Marzouk, Professor of Phytochemistry and Plant Chemosystematics, with respective voucher numbers for *Rheum rhabarbarum* L. (M180) and *Aloe vera* (L.) (M181).

### Chemical and biochemical analysis

#### Chemical

Oleic acid 90%, polysorbate 20 (Tween 20), Sodium Glycocholate 97.5%, Sodium Cholate 99%, and Distilled water (de-ionized), all chemicals were purchased from Alfa Aesar, Karlsruhe, Germany, and used with no further purification. Chemicals used for biochemical analysis were the Bovine albumin standard, purchased from Stanbio Laboratory (Texas, USA); Commasie brilliant blue G-250, purchased from Sigma (Sigma Chemical Co.); P- nitroanisole (purity 97%), acquired from Ubichem Ltd. (Ham pshire); and nicotinamide ademine dinucleotide phosphate (reduced form, NADPH), got from BDH chemicals Ltd. (Poole, England). The rest of the chemicals were of high quality purchased from commercial local companies and used without further purification.

#### Synthesis of plant extracts

##### Synthesis of plant extracts before nanoformulations

Both *A. vera* leaves and *R. rhabarbarum* stems were separately washed with distilled water twice and attained to be dehydrated. After complete dryness for three days at 50 °C in a vacuum oven, the plants were ground to a fine powder and washed several times with distilled water. About 250 g of each plant was placed in a beaker containing about 600 ml of 10% ethanol (v/v). The beaker was transferred to a hotplate and the temperature was raised to 50 °C for 3 h with occasional mixing using a glass rod (every 10 min) with flipping the flakes up and down to achieve good extraction. Afterward, the beaker was attained to cool to room temperature and then cooled at a temperature of 5–10 °C for two hours. The cooled beaker was filtered several times using a cotton tissue and then filtered using a Whatman filter paper; the supernatant was re-concentrated using a vacuum rotary evaporator to 50 ml. The net solution was kept in a dark glass bottle and kept at a temperature of 5–10 °C. For easier concentration manipulation and definite solid-content weight quantification, a small volume of 15 ml (pre-weighted) was re-concentrated using freeze drier utile complete solvent evaporation and collection of solid contents.

##### Preparation of extract-nanoemulsions

Preparation of nanoemulsions of the plant extracts was done according to a previous portocol^65^ with little modifications, as follows: 2 gm of oleic acid, placed in a 50 ml beaker, was heated to 40 °C (solution I). On another 50-ml beaker, 2.5 ml solution of Tween 20 and 15 ml of concentrated extract were added to a well-stirred mixture consisting of 0.7 gm sodium glycolate, and 0.7 gm sodium taurocholate dissolved in 10 ml water, a portion-wise addition, and then the overall mixture heated to the same temperature 40 °C (solution 2). Solution I is then poured into solution II at the same temperature to get a solution of a clear nanoemulsion at 40 °C, which in turn is dispersed using an ultrasonic probe sonicator for 20 min at 500 W with the addition of 100 ml ice cold water. Mannitol was added to the dispersion as a cry protectant and then lyophilization was done to get a semi-solid substance.

#### Phytochemical analyses of plant extract

High-performance liquid chromatography (HPLC) analyses were done using an Agilent 1260 series. The separation process was carried out using the Eclipse C18 column (4.6 mm × 250 mm i.d., 5 μm). The mobile phase was comprised of water (A) and 0.05% trifluoroacetic acid in acetonitrile (B) at a flow rate of 0.9 ml/min. Such phase was programmed consecutively in a linear gradient as follows: 0 min (82% A); 0–5 min (80% A); 5–8 min (60% A); 8–12 min (60% A); 12–15 min (82% A); 15–16 min (82% A); and 16–20 (82%A). The multi-wavelength detector was monitored at 280 nm. The injection volume was 5 μl for each of the sample solutions. The column temperature was maintained at 40 °C.

#### Characterization of nanoemulsion

##### Droplet size and Zeta Potential

The hydrodynamic radius of the synthesized extract nanoemulsions and polydispersity index (PDI) were done by dynamic light scattering (DLS) at a fixed angle of 173° at room temperature. Zeta potential or the surface charge was measured by the frequency shift of scattered light at a scattering angle of 12°. Moreover, PDI, Radius, and Zeta potential were investigated by a Zetasizer nano Zs analyzer (Malvern instruments) at the Egyptian Petroleum Research Institute (EPRI), Cairo, Egypt. About 5–10 mg of each powder was dispersed in 10 mL of distilled water at a temperature of 25 °C.

##### NLC surface morphology by transmission electron microscope (TEM)

The morphology and internal structure visualization of obtained nanoemulsions were investigated using field transmission electron microscopy (HR-TEM, JSM-7100F) at EPRI. Images were recorded with JEOL JEM-2100-115 a high-resolution transmission electron microscope with an accelerating voltage of 200 kV. Nearly 1 µL of NLCs was diluted with double distilled water (1:200) and placed on a 200 mesh carbon-coated grid and attained for two min. and the excess liquid was disposed of by filter paper. One to two drops of 2% (w/w) phosphotungstic acid (PTA) were added to the grid for 10 s to achieve negative staining, the excess PTA was removed via adsorption on a filter paper.

### Tick

Adult males and engorged females of *H*. *dromedarii* were collected from places around infested camels in Toukh (35 km north Cairo: 30° 21′ 11.6″ N and 31° 11′ 31.5″ E), Qalyubia Governorate, Egypt. Ticks were transferred to the laboratory in plastic cups covered by a piece of cotton net gauze. Morphological identification was performed^66^.

N.B. This study involved the treatment of ticks and did not involve live vertebrates. All experiments were accomplished in agreement with the relevant guidelines and regulations of the Ethical Committee of the Faculty of Veterinary Medicine, Benha University, Egypt (BUFVTM 02–10-22).

### Adult immersion tests

#### Adulticidal effect

An in-vitro adult immersion test (AIT) was used to evaluate the toxicity of plant extracts against *H. dromedarii* in line with a previously described protocol^67^. Five and six concentrations were diluted in distilled water for each plant extract and its nanoemulsions, respectively. Ten active males were immersed for 60 s in a 100 ml solution at each concentration.

Three replicates were used for each concentration (30 ticks/ concentration) and the control group was treated with distilled water. After immersion, ticks were added to a Petri dish with filter paper (Whatman no. 1) and kept at 27 ± 2 °C and 80 ± 5% relative humidity. Tick mortalities were checked up to 15 days post-treatment (PT) and recorded as dead when no reaction was shown after stimulation with a fine brush.

#### Insect growth regulating effect

Tests were carried out to determine the efficacy of plant extracts before and after nanoformulations against engorged females of *H. dromedarii*, according to a previously described protocol^18^ with a slight modification. Five concentrations were freshly prepared in distilled water. Thirty ticks were individually weighted and treated as mentioned in the previously mentioned AIT protocol for each concentration. Each immersed tick was kept uprightly in a labeled vertical test tube covered with a cotton plug at 27 ± 2 °C and 80 ± 5% relative humidity. The weight of the egg mass was measured and the number of hatched eggs was counted.

### Biochemical and enzyme assay analyses

#### Apparatus

Ticks were treated with the LC_50_ of each tested material and homogenized for biochemical analysis in a chilled glass Teflon tissue homogenizer (ST–2 Mechanic-Preczyina, Poland). After homogenization, supernatants were kept in a deep freezer at -20 °C till used for biochemical assays. A double-beam ultraviolet/ visible spectrophotometer (Spectronic 1201, Milton Roy Co., USA) was used to determine the optical density of the colored substances or metabolic compounds.

#### Preparation of ticks for analyses

Treated ticks were homogenized in distilled water (50 mg /1 ml); homogenates were centrifuged at 8000 r.p.m. for 15 min at 2 °C in a refrigerated centrifuge. After that, the deposits were discarded and the supernatant (enzyme extract) was stored at < 0 °C for less than a week until used. All experiments contained three replicates (tick homogenates) and the results of biochemical determinations were pooled in triplicates^68^.

Total proteins^69^ and total carbohydrates were assessed in an acid extract of the sample by the phenol-sulphuric acid reaction^70^, extracted and prepared for the assays^71^. Acetylcholinesterase (AchE) activity was measured using acetylcholine bromide (AchBr) as a substrate^72^; Alpha esterases (α-esterases) were determined using α-naphthyl acetate as a substrat^73^. Determination of amylase activity was also revealed^68^.

### Data analyses

The data were analyzed through SPSS V23 (IBM, USA) to perform the one-way analysis of variance (ANOVA) (Post Hoc/Tukey's HSD (honestly significant difference) to compare the significant difference within and between groups and the Probit analyses to calculate the lethal concentration (LC) and time (LT) values. All significant levels were set at P<0.05.

The mortality data were corrected^74^ according to the following equation:

$${\text{Corrected Mortality}}\% \, = \, \left( {{\text{MT}}\% - {\text{ MC}}\% } \right)/ \, \left( {{1}00 - {\text{MC}}\% } \right){\text{ X 1}}00$$

MT: mortality of the treated group; MC: mortality of the control group.

The relative toxicities^46^ and the toxicity indices were determined^75^ for a comparison of the tested extracts, where the most toxic plant extract had given 100 units on the toxicity index scale.

$${\text{Relative toxicity}} = {\text{ LC}}_{{{5}0}}\, \left( {{\text{or LC}}_{{{9}0}} } \right){\text{ of the least toxic plant extract}}/{\text{LC}}_{{{5}0}} \,\left( {{\text{or LC}}_{{{9}0}} } \right){\text{ of each tested plant extract}}.$$

$${\text{Times potency}} = {\text{ LT}}_{{{5}0}}\, {\text{of the least toxic plant extract}}/{\text{LT}}_{{{5}0}}\, {\text{of each tested plant extract}}$$

$${\text{Toxicity index}} = {\text{ LC}}_{{{5}0}}\, {\text{of the most toxic plant extract }} \times { 1}00/{\text{LC}}_{{{5}0}}\, {\text{of each tested plant extract}}.$$

### Ethical approval

The protocol of this work was approved by the Ethical Committee related to the Faculty of Veterinary Medicine at Benha University, Egypt (BUFVTM 02-10-22).

## Results and discussion

*Hyalomma* ticks have economic importance^76^. Previous studies indicated that *H. dromedarii* had acquired resistance against the commercially and widely used acaricides, Deltamethrin (Butox®)^10^ and Phoxim® (50%, an analogous dimethyl ester, C_12_H_15_N_2_O_3_PS) in Egypt^11^, in Qalyubia Governorate, Egypt, the same locality of the present study. Consequently, searching for eco-friendly acaricides is a pressing need^12,16,67^. Besides their pesticidal effects, botanicals have fungicidal, bactericidal, and antioxidant properties; therefore, they are used in medicine and cosmetics^15,24,32,49^. There are few studies on the efficacy of herbal extracts against *H. dromedarii*^10,78–81^ and very rare studies tested their effect on its reproductive potential^50^. This study evaluated the innovative acaricidal efficacy of ethanolic extracts of *A. vera* and *R. rhabarbarum* and their nanoemulsions against males and engorged females of *H. dromedarii* to break its life cycle*.*

### Phytochemical analyses

#### HPLC of the ethanol extracts

To identify the components presented in the ethanol extracts of *A. vera* and *R. rhabarbarum* extracts in this study, HPLC analysis was carried out with 19 standard polyphenols (Table 1). Active ingredient polyphenols were revealed for *A. vera* (Table 2) and *R. rhabarbarum* (Table 3) and the HPLC analysis indicated that both ethanol extracts were enriched with polyphenolic and flavonoid active ingredients that may give a good interpretation of their acaricidal and IGR effects. Alike findings were reported^28,35,81^. The most active ingredients identified by GC-MS analysis in *A. vera* gel extract were Terpene and Sesquiterpene hydrocarbons^82^. Moreover, a combination of active ingredients in the extract could synergistically increase the biological activity of the extract^19,20,51^.

**Table 1:** Standard polyphenols used for The High-Performance Liquid Chromatography (HPLC) analyses.

Standard	Conc. (µg/ml)	Area
Gallic acid	15	171.65
Chlorogenic acid	50	373.44
Catechin	75	291.29
Methyl gallate	15	239.43
Coffeic acid	18	241.80
Syringic acid	17.2	208.30
Pyro catechol	40	523.90
Rutin	61	445.91
Ellagic acid	120	327.76
Coumaric acid	20	710.86
Vanillin	12.9	338.87
Ferulic acid	20	324.86
Naringenin	30	259.83
Daidzein	35	491.37
Querectin	40	310.98
Cinnamic acid	10	459.44
Apigenin	50	619.44
Kaempferol	60	507.81
Hesperetin	20	334.36

**Table 2:** The High-Performance Liquid Chromatography (HPLC) of *Aloe vera.*

	Area	Conc. (µg/ml = µg/25 mg )	Conc. (µg/g )
*Aloe vera*			
Gallic acid	126.25	11.03	441.29
Chlorogenic acid	16.94	2.27	90.73
Catechin	55.10	14.19	567.51
Methyl gallate	37.35	2.34	93.59
Coffeic acid	0.00	0.00	0.00
Syringic acid	17.59	1.45	58.09
Pyro catechol	5.14	0.39	15.69
Rutin	2.49	0.34	13.64
Ellagic acid	18.65	6.83	273.18
Coumaric acid	69.46	1.95	78.17
Vanillin	0.00	0.00	0.00
Ferulic acid	85.29	5.25	210.02
Naringenin	75.04	8.66	346.56
Daidzein	20.36	1.45	58.00
Querectin	7.17	0.92	36.88
Cinnamic acid	3.57	0.08	3.11
Apigenin	6.05	0.49	19.53
Kaempferol	0.00	0.00	0.00
Hesperetin	19.74	1.18	47.23

**Table 3:** The High-Performance Liquid Chromatography (HPLC) of *Rheum rhabarbarum.*

	Area	Conc. (µg/ml = µg/25 mg )	Conc. (µg/g)
*Rheum rhabarbarum*			
Gallic acid	386.46	33.77	1350.86
Chlorogenic acid	43.17	5.78	231.19
Catechin	628.88	161.92	6476.79
Methyl gallate	6.90	0.43	17.28
Coffeic acid	19.86	1.48	59.14
Syringic acid	0.00	0.00	0.00
Pyro catechol	1.29	0.10	3.93
Rutin	0.00	0.00	0.00
Ellagic acid	0.00	0.00	0.00
Coumaric acid	498.95	14.04	561.51
Vanillin	0.00	0.00	0.00
Ferulic acid	638.85	39.33	1573.21
Naringenin	241.05	27.83	1113.26
Daidzein	759.38	54.09	2163.61
Querectin	450.33	57.92	2316.96
Cinnamic acid	368.19	8.01	320.55
Apigenin	89.90	7.26	290.25
Kaempferol	0.00	0.00	0.00
Hesperetin	75.48	4.51	180.59

This study revealed that *A. vera* extract was rich in many active ingredients, such as catechin, gallic acid, naringenin ellagic acid, and ferulic acid (567.51, 441.29, 346.56, 273.18, and 210.02 µg/g, respectively) and good amounts of methyl gallate, chlorogenic acid, coumaric acid, syringic acid, daidzein, hesperetin, querectin, apigenin, pyro catechol, rutin, and cinnamic acid (93.59, 90.73, 78.17, 58.09, 58.00, 47.23, 36.88, 19.53, 15.69, 13.64 and 3.11 µg/g, respectively). In contrast, kaempferol, vanillin, and coffeic acid had no participation from *A. vera* extract (Table 2).

In contrast to our findings, another study implied that *A. vera* contains acemannan, valoin a and b, homonataloin, Aloe emodin, etc. in its latex. Furthermore, aloe leaf extract contains 99% water and 75 active compounds including vitamins, minerals, amino acids, and enzymes (peroxidase, lipase, cellulase, catalase, carboxypeptidase, bradykinase, amylase, and alkaline phosphatase)^83^.

Similar to the contents of *A. vera* in the present study, *R. rhabarbarum* contained catechin, querectin, daidzein, ferulic acid, gallic acid, and naringenin (6476.79, 2316.96, 2163.61, 1573.21, 1350.86, and 1113.26 µg/g, respectively); whereas, coumaric acid, cinnamic acid, apigenin, chlorogenic acid, hesperetin, coffeic acid, methyl gallate, and pyro catechol have good abundance (561.51, 320.55, 290.25, 231.19, 180.59, 59.14, 17.28, and 3.93 µg/g, respectively). On the other hand, rutin, vanillin, kaempferol, ellagic, and syringic acids had no abundance in *R. rhabarbarum* (Table 3).

Phytochemicals in Rhubarbs in another study included stilbenes, anthraquinones, and flavonoids^59^. The phytochemical characterization of the extracts of *Aloe arborescens* enabled the identification of the presence of condensed and water-soluble tannins, besides anthraquinones, including aloeresin, aloenin, aloin A and B, homonataloin, and 4′-O-glucosylisoaloeresin. Water-soluble tannins were the main components of the extracts with acaricidal activity^84^. The presence of the phenolic contents may play an important role in the elimination of ticks and mites through their action on the GABA receptor and the octopamine receptor^85^.

Against *H. dromedarii* in Egypt*, Saussurea costus* extract contains mainly sesquiterpene, fatty acid esters, phenols, and acyclic hydrocarbons which might explain its pesticidal effect^86^. A similar study showed that myrrh is slightly more effective than ginger against *H. dromedarii* and this may be due to its high contents of flavonoids and phenols^10^. The total phenolic and flavonoid contents of nine aqueous plant extracts explain their acaricidal efficacy; *Ricinus communis* possessed the highest total phenolic contents (95.50 ± 0.17 mg/g), while *Quercus cortex* contained the highest flavonoid contents (70.78 ± 0.17 mg QE/g)^77^.

#### Physicochemical properties of nanoemulsion- plant extracts

##### Dynamic light scattering (DLS) and Zeta potential (z.p)

According to the "Brownian motion", when a small suspended particle moves through a liquid solution, it will undergo randomized motion because of the collisions by the molecules themself and; consequently, particles. Furthermore, DLS is a very reliable and powerful technique to study the diffusion attitude of the small to relatively large particles in a solution depending on the particle’s size (hydrodynamic radii) and shape variations. After size and shape data collection, the data were analyzed internally by the device to obtain the degree of the heterogeneity, or what so-called poly dispersity index (PDI), of the solution based on size variation, which may occur as a result of agglomeration or aggregation. The International Organization for Standardization (ISOs) declared that if the values of PDI were less than 0.05, the solution under investigation is more likely to be monodispersed or homogenous, while values > 0.7 indicated that the solution is commonly of broad size or polydisperse particle distribution^27,87^. In this study, DLS of the prepared *A. vera* extract nanoemulsion pretended particle size of 196.7 nm with pdi of 0.300, confirmed that the synthesized *A. vera* extract nanoemulsion had a good particle size with polydisperse particle distribution. Like *A. vera* extract nanoemulsion, *R. rhabarbarum* nanoemulsions showed a slightly larger average size of 291nm with little reduction in the pdi value of 0.239, but it was still fit to be described by" polydispersed” particle distribution (Table 4).

**Table 4:** DLS and Zeta Nanoemulsion.

Nano sys	DLS/nm	Zeta/mV
*Aloe vera*	196.7	− 29.1
*Rheum rhabarbarum*	291	− 53.1

The stability of the colloidal system related to the dispersion of solid materials in the liquid was monitored with zeta potential. It is caused by the net electrical charges in a specific region in a slipping plane depending on the location of such plane. Zeta potential magnitude indicates the degree of electrostatic repulsion forces between the same adjacent charged particles in the dispersion. For small particles like molecules, a higher zeta potential will promote stability, i.e., dispersion will resist aggregation, and vice versa when the zeta potential is small enough to make the attractive forces exceed the repulsion forces, i.e., particles become closer to each other and the dispersion will be flocculated^34^. Consequently, colloids with high values of zeta potential, even negative or positive, are electrically stabilized while colloids with low zeta potential values have a large tendency to flocculate or coagulate. It is interesting that the results of this study showed values of zeta potential of *R. rhabarbarum* extract nanoemulsion of −53.1 mV. The larger negatively charged numerical value reflects the higher repulsion forces exerted on the system due to the similar negative charges. Increasing the number and concentrations of polyphenols and flavonoids in the extract promoted extra negative charges because of a wide spread of hydroxyl groups. From this point of view, *A. vera* nanoemulsion extract contained less concentration of polyphenolic active ingredients that may affect the net charge or zeta potential to be −29.1 mV. Comparatively, the zeta potential of the nanoemulsion of *R. rhabarbarum* is much greater than that of *A. vera* indicating that *R. rhabarbarum* nanoemulsin had excellent stability. In the meantime, both nanoemulsions have good stability attitudes^88^.

##### Transmission electron microscope (TEM) findings of plant extract-loaded nanoemulsions

**Figure 1:**
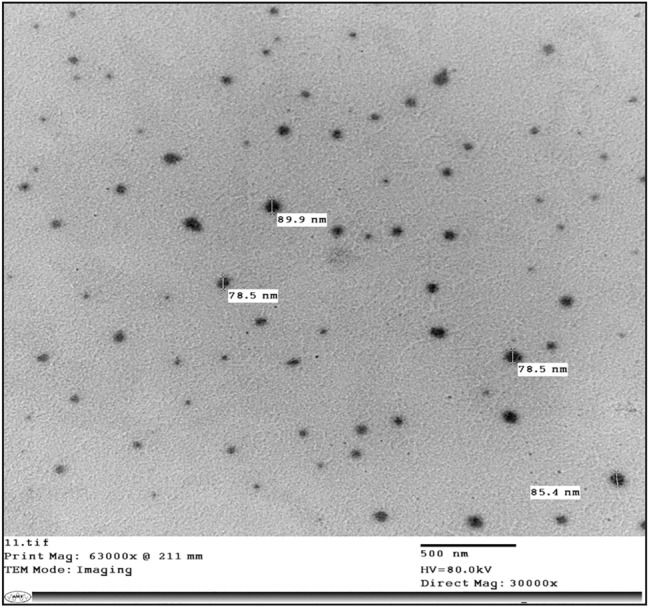
Transmission electron microscope (TEM) of *Aloe vera* extract-nanoemulsion.

**Figure 2:**
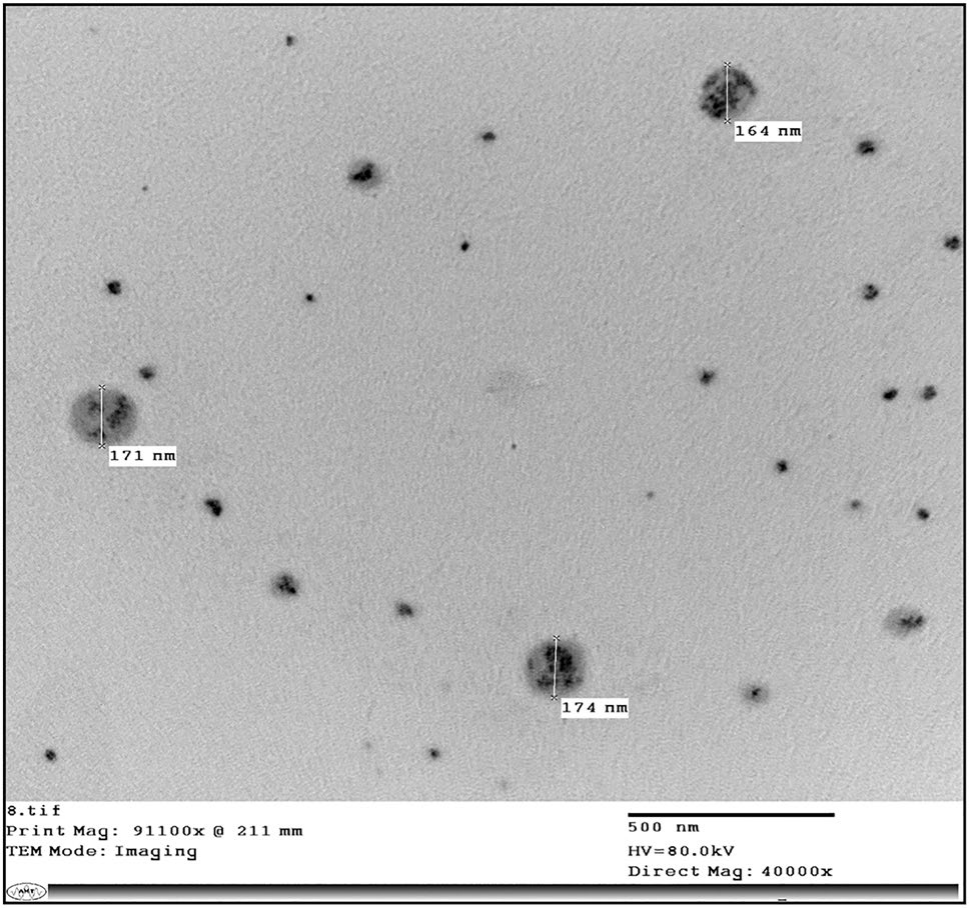
Transmission electron microscope (TEM) of *Rheum rhabarbarum* extract-nanoemulsion.

The transmission electron microscope is one of the most important analyses to confirm the internal structure of nanoemulsions prepared in this study (Figs. 1 and 2). Nanoemulsions of *R. rhabarbarum* and *A. vera* extracts showed regular and spherical shapes in various sizes (less than 100nm); whereas that of *R. rhabarbarum* presented a slightly higher size near 200 to 600nm confirming the polydispersity depicted by PDI findings. The revealed size in this study came along with data obtained by zeta sizer; the results of DLS measure the average size, not definite particle size like that of TEM^27^.

### Acaricidal activity of the plant extracts against *H*.* dromedarii*

The mortality data of the present study showed a time and concentration-dependent relationship; similar studies were recorded^12,67^. Three days post-treatment (PT) with 25% of* A*. *vera* and *R. rhabarbarum*, the mortality percentage (MO%) reached 50 and 53.6%, respectively. Five days PT, MO% reached 61.5 and 65.4%, respectively. Meanwhile, 15 days PT, MO% reached 88.5% and 96.2%, respectively (Table 5). Three days PT, the LC_50_ and LC_95_ values of *A*. *vera* were 9.8 and 25.6%; whereas those of *R. rhabarbarum* were 8.9 and 24.8%, respectively (Table 6). Such values five days PT were 7.8 and 22.9% for *A*. *vera* and 7.1 and 21.4% for *R. rhabarbarum*, respectively (Table 7). The toxicity index values were 90.8% and 100%, at three days PT, and reached 91.02 and 100%, respectively, at five days PT (Tables 6 and 7). Meanwhile, LT_50_ values PT with 25% and 3.5% of* A*. *vera* were 1.86 and 6.09 days; while those of *R. rhabarbarum* were 1.65 and 5.09 days, respectively (Table 8).

**Table 5:** The adulticidal effect of ethanol extracts against *Hyalomma dromedarii* males.

Plant extracts	Conc.%	The corrected motility of means ± SD Day post-treatment							
		Day1	Day2	Day3	Day5	Day7	Day9	Day12	Day15
Control		0.00^b^	0.00^b^	0.00^b^	0.00^b^	0.00^b^	0.00^b^	0.00^b^	0.00^b^
*Aloe vera*	**1.5**	16.7 ± 0.33^d^	20.0 ± 0.6^d^	23.3 ± 0.6^d.d^	33.3 ± 0.6 ^c.d^	43.3 ± 0.6^b.c^	50.0 ± 0.0^a.b^	53.3 ± 0.6^a.b^	60.0 ± 0.0^a^
	**3**	20.0 ± 0.0^d^	30.0 ± 0.0^d.c^	33.3 ± 1.15^d.c^	40.0 ± 0.6^b,c^	53.3 ± 1.15^a,b^	60.0 ± 0.0^a^	63.3 ± 0.6^a^	70.0 ± 0.0^a^
	**6**	26.7 ± 0.33^d^	36.7 ± 0.3^d^	46.7 ± 1.15^c.d^	50.0 ± 0.6^b.c.d^	60.0 ± 0.0^a.b.c^	66.7 ± 0.6^c.d^	70.0 ± 0.0^a.b^	73.3 ± 0.6^a^
	**12**	33.3 ± 0.6^c^	43.3 ± 0.0^c^	50.0 ± 0.2^c^	60.0 ± 16.1^c^	70.0 ± 0.6^c^	76.7 ± 0.6^c^	80.0 ± 0.0^c^	83.3 ± 0.6^c^
	**25**	41.4 ± 0.6^d^	46.4 ± 1.0^c.d^	50.0 ± 1.15^c.d^	61.5 ± 1.12^b.c^	73.1 ± 0.6^a.b^	77.1 ± 0.0^a.b^	84.7 ± 0.6^a.b^	88.5 ± 0.0^a^
*Rheum rhabarbarum*	**1.5**	16.7 ± 0.6^d^	23.3 ± 1.2^d^	26.7 ± 0.6^c.d^	36.7 ± 0.9^b.c.d^	46.7 ± 0.6^a.b.c^	50.0 ± 0.0^a.b^	53.3 ± 0.6^a.b^	60.0 ± 0.0^a^
	**3**	20.0 ± 0.6^d^	33.3 ± 1.2^d^	40.0 ± 1.0^d.c^	43.3 ± 1.5^b.c.d^	56.7 ± 0.6^a.b.c^	63.3 ± 0.0^a.b^	66.7 ± 0.6^a^	73.3 ± 0.0^a^
	**6**	30.0 ± 1.0^d^	43.3 ± 0.6^d^	50.0 ± 1.0^c.d^	53.3 ± 0.6^b.c.d^	63.3 ± 0.6^a.b.c^	70.0 ± 0.0^a.b^	73.3 ± 0.6^a^	76.7 ± 0.6^a^
	**12**	33.3 ± 1.15^d^	43.3 ± 0.6^d^	53.3 ± 1.2^d^	63.3 ± 0.6^c.d^	73.3 ± 0.6^a.b.c^	76.7 ± 0.6^a.b^	83.3 ± 0.6^a.b^	86.7 ± 0.6^a^
	**25**	44.8 ± 0.6^c^	50 ± 1.2^b.c^	53.6 ± 1.0^b.c^	65.4 ± 2.0^b,c^	76.9 ± 1.0^a.b^	80.8 ± 0.6^a.b^	92.3 ± 0.6^a^	96.2 ± 0.6^a^

Means followed by the same letter in the same row are not significantly different from ANOVA (*p* > 0:05).

*Conc.%* Concentration%

**Table 6:** Lethal values (LC) of ethanol plant extracts of *Aloe vera* and *Rheum rhabarbarum* against *Hyalomma dromedarii* males, three days post-treatment.

Plant extract	*Aloe vera*			*Rheum rhabarbarum*		
	LC_50_ Lower upper	LC_90_ Lower upper	LC_95_ Lower upper	LC_50_ Lower upper	LC_90_ Lower upper	LC_95_ Lower upper
	9.8 7.46 14.99	22.1 16.36 37.12	25.6 18.79 43.49	8.9 4.02 39.50	21.3 15.01 32.40	24.8 17.41 38.51
Chi	8.421			10.238		
Df	4			4		
Sig	0.077^a^			0.037a		
Regression	Y = 1.18 + 0.12*X			Y = 1.08 + 0.12*X		
R^2^	0.641			0.591		
Relative toxicity	1.0			1.1		
Toxicity index	90.8			100		
Reference	*Aloe vera*					

Concentration%

**Table 7:** Toxicity of ethanol plant extracts of *Aloe vera* and *Rheum rhabarbarum* against *Hyalomma dromedarii* males, five days post-treatment.

Plant extract	*Aloe vera*			*Rheum rhabarbarum*		
	LC_50_ Lower upper	LC_90_ Lower upper	LC_95_ Lower upper	LC_50_ Lower upper	LC_90_ Lower upper	LC_95_ Lower upper
	7.8 5.90 11.47	19.6 14.72 31.69	22.9 17.09 37.55	7.1 2.89 16.41	18.3 10.72 60.89	21.4 12.43 93.56
Chi	9.411			11.060		
Df	4			4		
Sig	.052a			.026a		
Regression	Y = 1.02 + 0.13*X			Y = 0.98 + 0.13*x		
R2	0.602			0.596		
Relative toxicity	1.0			1.1		
Toxicity index	91.02			100		
Reference	*Aloe vera*					

Concentration%

Relative toxicity = LC_50_ of the least toxic compound/LC_50_ of the tested compound.

Toxicity index = LC_50_ of the most toxic compound × 100/LC_50_ of the tested compound.

**Table 8:** Lethal time (LT) value (per day) and time potency of *Aloe vera* and *Rheum rhabarbarum* post-treatment of *Hyalomma dromedary* males.

Plant extracts	Concentration%											
	25			12			6			3.5		
	LT_50_ Lower upper	LT_90_ Lower upper	Time potency	LT_50_ Lower upper	LT_90_ Lower upper	Time potency	LT_50_ Lower upper	LT_90_ Lower upper	Time potency	LT_50_ Lower upper	LT_90_ Lower upper	Time potency
*Aloe vera*	1.86 (0.99–2.68)	19.70 (12.02–51.91)	1.0	2.63 (1.61–3.64)	28.62 (16.35–87.63)	1.0	3.99 (2.57–5.69)	62.34 (28.05- 389.57)	1.0	6.09 (4.38–8.95)	75.37 (34.04–425.79)	1.0
*Rheum rhabarbarum*	1.65 (0.94–2.31)	12.77 (8.66–25.29)	1.1	2.45 (1.53–3.35)	22.48 (13.82–56.28)	1.07	3.17 (1.85–4.55)	52.93 (24.41–321.19)	1.1	5.09 (3.62–7.15)	59.36 (28.77–274.71)	1.2
Reference	*Aloe vera*											

Times potency = LT_50_ of the least toxic plant extract/ LT_50_ of each tested plant extract.

Parallel to our findings*,* the hydroethanolic extract of the leaves of *Aloe rupestris* induced an acricidal effect (66.6%) against the cattle tick, *Rhipicephalus turanicus* (Acari: Ixodidae) at a concentration of 20% (200 mg/mL)^89^. In addition, aqueous extracts (5%) of *A. vera*, garlic, *Allium sativum,* and ginger*, Zingiber, officinale,* against the brown dog tick, *Rhipicephalus sanguineus*, showed acaricidal effect (100%) against females, males, and larvae and showed cuticular damage along with breaching and loss of homogeneity of both epicuticle and endocuticle^90^. The findings of *A. vera* in this study came along with some other studies as it has a great potential for development as a botanical acaricide against the carmine spider mite, *Tetranychus cinnabarinus* (LC_50_ values of the acetone extract were 0.614 and 0.099 mg/ml, 48 h and 72 h PT, respectively)^91^ and the females of the two-spotted spider mite, *Tetranychus urticae* (LC_50_ values of petroleum ether, methanol, ethanol, and acetone extracts reached 1.279, 0.953, 0.667, and 0.446%, respectively)^92^.

Very recent and similar studies revealed the efficacy of other botanicals as acaricides. Complete mortalities of *H. dromedarii* and *Rhipicephalus (Boophilus) annulatus* were recorded PT with 25 mg/mL of *Araucaria heterophylla* and *Commiphora molmol* extracts for seven days. The LC_50_ values PT of *H. dromedarii* with the methanol and hexane extracts were 1.13 and 1.04 mg/mL and 1.47 and 1.38 mg/mL, respectively; whereas such values against *R. annulatus* were 1.09 and 1.41 as well as 1.55 and 1.08 mg/mL, respectively^28^. The influence of the ethanol extracts (25%) of costus, *Vitex castus*, and, *Z. officinale,* against *H. dromedarii* demonstrated that MO% 15 days PT reached 80.8 and 84.7%, respectively; their LC_50_ values, five days PT, were 10.50 and 9.60%, respectively; and their toxicity indices reached 91.43 and 100.00%, respectively. On the other hand, their LT_50_ values PT with 25% were 2.6 and 2.5 days, respectively^78^. Moreover, the adulticidal effect of olive, *Olea europaea* L, oil against *H. dormidarii* males reached 83.33%, 15 days PT with 25%, and its LT_50_ and LT_95_ were 5.161 and 17.072 days, respectively, whereas its LC_50_ and LC_95_ values were 12.715 and 46.386%, respectively, 12 days PT^50^.

Analogs to our findings, another species of *R. palmatum* extract and five of its isolated anthraquinones possessed insecticidal activity against the brown planthopper*, Nilarparvata lugens,* and the northern armyworm*, Mythimna separate*. A high amount of *R. palmatum* compounds (82.87 mg/g) were detected in its acetone extract inducing stronger insecticidal effect than those of the aqueous and ethanol extracts (yields = 8.84, 37.17, and 36.92%, respectively). Amongst the isolated compounds in such study, emodin displayed very high and moderate insecticidal activity, as its LC_50_ values were 84.30 and 548.74 μg/mL, respectively). A similar pattern was recorded for toosendanin (LC_50_= 89.34 and 418.24 μg/mL, respectively^93^. In contrast to our superior acaricidal effect, fractions of *R. palmatum* root extract and anthraquinone (aloe-emodin, emodin, chrysophanol, and physcion) showed no acaricidal activities against the mite, *T. urticae*^94^. Different outcomes might be related to using different pest species and extracts. Moreover, *R. palmatum* has fungicidal^93,94^ and herbicidal activitie^94^.

Similar adulticidal efficacies were reported after treating the engorged females of *H. dromedarii,* collected from the same locality of the present study and treated with the same *in vitro* immersion bioassays, and a 100% lethal effect was recorded at 8 h PT with 2% rose Bengal (a photosensitizer) and 24 h PT with 2.5%, ivermectin (a macrocyclic lactone). Their LT_50_ values were 0.92 and 2.63 h PT with 2% rose bengal and 2.5% ivermectin, respectively, whereas their LC_50_ values were 0.08 and 0.35%, respectively, and their LC_95_ values reached 1.45 and 30.07%, respectively^18^. An analogous study specified that a complete adulticidal effect was recorded PT of the engorged females of *H. dromedarii* with 4% safranin and tetramethrin, for 8 and 48 h, respectively. LC_50_ values eight and 24 h PT were 0.08 and 0.03% as well as 0.78 and 0.20%, respectively. In addition, LT_50_ of safranin and tetramethrin were 0.80 and 2.17 h, respectively, PT with 4%^67^. Against *H. dromedarii,* Methylene blue was the most effective material 3 days PT (LC_50_=127 ppm) followed by safranin, field stain, rhodamine 6G, phthalocyanine, echinochrome, ribofavin, and chlorophyllin (LC_50_= 209, 251, 271, 303, 324, 332, and 362 ppm, respectively). Their LT_50_, values reached 45, 87, 96, 72, 129, 115, 131, and 137 h, respectively, PT with 240 ppm^11^.

A comparable study revealed the acaricidal activity of *Citrus limetta* seed oil against the cattle tick, *Rhipicephalus microplus* (LC_50_ = 2.87 and 3.96% PT of larvae and adults, respectively) and 100% MO were reached PT with 12.5%^95^. Furthermore, the crude extract along with water and petroleum ether fractions of *Areca (A.) catechu* seeds were effective against cypermethrin-resistant *R. (R.) microplus*^53^. In addition, *Saussurea costus* as methanol and hexane extracts of effectively controlled cattle and camel ectoparasites; MO% of *H. dromedarii*, seven days, PT with 12.5 and 25 mg/ml was 100 and 90% (LC_50_ = 1.37 and 2.33 mg/ml, respectively). In the meantime, such values against *R. (B) annulatus* were 100 and 93.33% coupled with 1.23 and 1.95 mg/ml, respectively^86^. A comparable acaricidal effect of an aqueous neem extract against *Sarcoptes scabiei var. cuniculi in vitro* and experimentally-infested rabbits was recorded^14^.

### Insect growth regulating effects of plant extracts against *H*.* dromedarii*

After treatment with *A. vera* and *R. rhabarbarum* in this study*,* the reproductive potential of engorged females was adversely affected when compared to that of the control group. After treatment with the highest concentration, 25%, egg production had ceased and engorged female weight reached 54.00 and 51.83 g, respectively (Table 9). Analogs result was recorded as the ethanol extract of *A. vera* effectively reduced egg production/ female of *T. urticae*, followed by acetone, methanol, and petroleum ether extracts by 96.0, 94.0, 85.0, and 83.0%, respectively (LC_50_ = 0.950, 1.406, 2.115, and 3.312%, respectively). Moreover, ethanol extract was the most effective repellent against *T. urticae* females^92^.

**Table 9:** The insect growth regulating the effect of plant extracts against *Hyalomma dromedarii* engorged females.

Ethanol plant extract	Conc. %	Number of Hatched eggs (Mean ± SD)	Hatchability%	Weight of Engorged females (g) (Mean ± SD)	Weight of Eggs (g) (Mean ± SD)
*Aloe vera*	Control	1203.33 ± 323.04^a^	96.7	77.33 ± 12.87^a^	38.43 ± 0.27^a^
	0.75	255.00 ± 87.81^b^	78.5	50.67 ± 2.16^b^	3.91 ± 2.25^b^
	1.5	113.33 ± 92.88^bc^	61.8	50.67 ± 2.16^b^	1.04 ± 1.37^b^
	3	30.83 ± 23.11^bc^	34.6	50.33 ± 2.25^b^	0.69 ± 1.02^b^
	12	0.00 ± 0.00^c^	0.0	50.83 ± 10.25^b^	00.00 ± 0.00^b^
	25	0.00 ± 0.00^c^	0.0	54.00 ± 4.00^b^	0.00 ± 0.00^b^
*Rheum Rhabarbarum*	Control	1203.33 ± 323.04^a^	96.7	77.33 ± 12.87^a^	38.43 ± 0.27^a^
	0.75	325 ± 113.40^b^	84.8	51.33 ± 3.01^b^	4.32 ± 1.02^b^
	1.5	137.50 ± 42.98^bc^	70.2	51.33 ± 3.01^b^	0.32 ± 0.31^b^
	3	17.66 ± 18.64^c^	33.3	51.33 ± 3.01^b^	0.32 ± 0.31^b^
	12	1.50 ± 1.94^c^	19.1	50.00 ± 11.08^b^	0.15 ± 0.18^b^
	25	0.00 ± 0.00^c^	0.0	51.83 ± 2.78^b^	0.05 ± 0.8^b^

A similar finding revealed a strong effect on the reproductive parameters represented by a marked decrease in the number of laid eggs PT of fresh and dry *Aloe arborescens* Mill. extracts (solvents pure ethanol, ethanol-dichloromethane binary mixture, and ethanol-dichloromethane-acetone ternary mixture, contained water-soluble tannins) against engorged females of *Rhipicephalus* (*Boophilus*) *microplus*^84^. A related finding revealed that *Citrus limetta* seed oil significantly (*p* < 0.001) lowered the oviposition rate, egg hatching, and reproduction efficiency of treated ticks of *R. microplus*^94^. Furthermore, Azadirachtin, a tetranortriterpene extracted from neem (LC50 = 0.47 ppm) adversely affected the fecundity and the development of the follicular epithelial cells of ovaries of female *N. lugens*, and the weight of the treated females was significantly reduced, 23, 40, and 64% PT with 0.1, 0.25, and 0.5 ppm, respectively^96^.

Few studies evaluated IGRs against *H. dromedarii* collected from the same locality of this study, olive oil induced IGR effect and adversely impacted the reproduction of the engorged females of *H. dromedarii* PT with 25% and adversely affected the number of hatched eggs (2.83 ± 2.31), hatchability (32.7%), as well as weights of females (52.50 ± 2.88 g) and egg masses (0.27 ± 0.27 g)^50^. Moreover, the number of survived females, ovipositing females, eggs per female, ticks laid hatched eggs, and hatched eggs were reduced PT with 0.01% of rose bengal (48.98%, 93.33%, 1854.53 ± 45, 97.5%, and 93.64%, respectively) and 0.02% of ivermectin (26.53%, 86.67%, 7661.27 ± 377, 87.80%, and 89.40%, respectively), respectively^18^. A comparable results were also recorded, PT with a low concentration (0.03%) of both safranin (75.0%, 89.13%, 3116 ± 70.26, 95.24%, and 81.00%, respectively) and tetramethrin (33.3%, 100.00%, 0.00, 100.00, and 100.00%, respectively^67^.

### Acaricidal activity of nanoemulsions against *H*.* dromedarii*

This study indicated the efficacy of nanoemulsions of *A. vera* and *R. rhabarbarum* against *H. dromedarii* PT with 15%; MO% reached 83.3 and 76.7%, respectively, three days PT; 86.6 and 90%, respectively, five days PT; and 100% for both extracts, 12 and nine days PT, respectively (Table 10). The LC_50_ and LC_95_ values were 4.2 and 17.67% PT for three days with nanoemulsions of *A. vera* and reached 3.5 and 17.4% PT with nanoemulsions of *R. rhabarbarum*, respectively (Table 11). Five days PT, the LC_50_ and LC_95_ values were 2.8 and 15.8% as well as 1.02 and 32.72%, respectively (Table 12). Regarding the LC_50_ values, the prepared nanoformulations enhanced the efficacy of the ethanol extracts of *A. vera* and *R. rhabarbarum* 2.3 and 2.5 times, three days PT (Table 11), and 2.8 and 7 times, five days PT (Table 12). *R. rhabarbarum* induced a superior effect and its toxicity index reached 100% (Tables 11 and 12). The LT_50_ values of *A. vera* and *R. rhabarbarum* were 0.898 and 0.839 days, respectively, PT with 15% and 1.75 and 1.34 days, respectively, PT with 3.5%. Nanoemulsions accelerated the speed of killing of ticks 2-4 times faster than the ethanol extracts (Table 13).

**Table 10:** Effect of the nanoemulsion of plant extracts post-treatment of *Hyalomma dromedarii* males.

Plant extracts	Extract concentration%	Mean mortalit% ± SD						
		Day1	Day2	Day3	Day5	Day7	Day9	Day12
Control		0	0	0	0	0	0	0
*Aloe vera*	0.5	10.0 ± 0.0^d^	16.67 ± 5.8^cd^	23.3 ± 5.8^bc^	33.3 ± 5.8^bc^	40.0 ± 0.0^bc^	46.6 ± 5.8^ab^	50.0 ± 5.8^a^
	0.9	23.33 ± 5.8^b^	33.3 ± 5.8^cd^	43.3 ± 5.8^bc^	50.0 ± 0.0^b^	53.3 ± 5.8^ab^	56.7 ± 5.8^ab^	66.7 ± 5.8^a^
	1.8	33.3 ± 11.5^c^	40.0 ± 10.0^b^	53.3 ± 11.5^a–c^	60.0 ± 0.0^ab^	63.3 ± 5.8^a^	70.0 ± 0.0^a^	70.0 ± 0.0^a^
	3.8	43.3 ± 5.8 ^d^	50.0 ± 10.0^cd^	60.0 ± 0.0^a^	66.67 ± 5.8^cb^	73.3 ± 5.8^ab^	76.67 ± 5.8^ab^	90.0 ± 0.0^a^
	8	46.67 ± 5.8^c^	53.3 ± 5.8^b,c^	66.67 ± 11.5^a–c^	73.33 ± 5.8^ab^	76.67 ± 11.5^ab^	80.0 ± 10.0^a^	86.67 ± 11.5^a^
	15	56.67 ± 15.3^c^	70.00 ± 10^bc^	83.33 ± 11.5^a,b^	86.67 ± 5.8^a^	93.3 ± 5.8^a^	96.7 ± 5.8^a^	100.0 ± 0.0^a^
*Rheum rhabarbarum*	0.5	10.0 ± 0.0^b^	20.0 ± 0.0^ab^	26.7 ± 5.8^a,b^	30.0 ± 0.0^ab^	40.0 ± 10.0^ab^	43.3 ± 20.8^a^	50.0 ± 17.3^a^
	0.9	23.33 ± 5.8^a^	33.3 ± 15.2^a^	50.0 ± 10.0^a^	53.3 ± 5.8^a^	56.7 ± 5.8^a^	60.0 ± 10.0^a^	66.7 ± 5.8^a^
	1.8	36.67 ± 11.5^c^	40.0 ± 11.5^bc^	53.3 ± 11.5^a,b^	60.0 ± 11.5^ab^	66.7 ± 11.5^ab^	73.3 ± 11.5^a^	76.7 ± 11.5^a^
	3.8	46.67 ± 5.8^d^	53.3 ± 11.53^bc^	66.7 ± 5.8^cd^	73.3 ± 0.0^bc^	76.7 ± 5.8^a–c^	80.0 ± 5.8 ^a.b^	86.7 ± 5.8^a^
	8	56.67 ± 11.5^a^	66.67 ± 5.8^a^	55.67 ± 24.8^a^	80.0 ± 10.0^a^	51.3 ± 42.1^a^	90.0 ± 10.0^a^	86.7 ± 11.5^a^
	15	63.3 ± 11.5^c^	73.3 ± 15.3^bc^	76.7 ± 5.8^a,c^	90.0 ± 10.0^ab^	96.7 ± 5.8^ab^	100.0 ± 0.0^a^	100.0 ± 0.0^a^

Means followed by the same letter in the same column are not significantly different by ANOVA (*p* > 0:05).

**Table 11:** Toxicity of nanoemulsions of *Rheum rhabarbarum* and *Aloe vera* post-treatment of *Hyalomma dromedarii* males three days post-treatment.

Plant extract	*Aloe vera*			*Rheum rhabarbarum*		
	LC_50_ Lower upper	LC_90_ Lower upper	LC_95_ Lower upper	LC_50_ Lower upper	LC_90_ Lower upper	LC_95_ Lower upper
	4.2 2.43–15.79	14.7 8.65 27.32	17.6 10.38 34.68	3.5 2.86 15.82	13.6 7.84 16.66	17.4 9.49 12.45
Chi	20.568			22.273		
Degree of freedom (df)	3			3		
Significance	0.001^a0^			0.000^a^		
Regression equation	Y = 0.65 + 0.13xX			Y = 0.58 + 0.13xX		
R squared (R^2^)	0.574			0.562		
Relative toxicity	1.0			1.2		
Toxicity index	83.3			100		
Reference	*Aloe vera*					
Relative toxicity* before and after nanoformulations*						
*(Reference: values before formulations)*	LC_50_	LC_90_	LC_95_	LC_50_	LC_90_	LC_95_
	2.3	1.5	1.5	2.5	1.6	1.4

Concentration:

Relative toxicity = LC_50_ of the least toxic compound/LC_50_ of the tested compound.

Toxicity index = LC_50_ of the most toxic compound × 100/LC_50_ of the tested compound.

**Table 12:** Toxicity of the tested nanoemulsion from *Aloe vera* and *Rheum rhabarbarum* five days post-treatment of *Hyalomma dromedarii* males.

Plant extract	*Aloe vera*			*Rheum rhabarbarum*		
	LC_50_ Lower upper	LC_90_ Lower upper	LC_95_ Lower upper	LC_50_ Lower upper	LC_90_ Lower upper	LC_95_ Lower upper
	2.8 1.92 5.82	12.9 7.23- 20.0	15.8 8.86 57.76	1.02 0.53 1.58	15.2 7.95 55.44	32.72 14.29 182.10
Chi	0.949			0.458		
Degree of freedom (df)	3			3		
Significance	0.917^a^			0.928^a^		
Regression equation	Y = 1*x + 0.02			Y = .8571*x + 0.1714		
R squared (R^2^)	0.975			0.968		
Relative toxicity	2.7			1.0		
Toxicity index	36.4			100		
Reference (lowest toxicity)	*Aloe vera*					
Relative toxicity* before and after nanoformulations*						
*(Reference: values before formulations)*	LC_50_	LC_90_	LC_95_	LC_50_	LC_90_	LC_95_
	2.8	1.5	1.4	7.0	1.2	0.7

Concentration: %; df: degree of freedom.

Relative toxicity = LC_50_ of the least toxic compound/LC_50_ of the tested compound.

Toxicity index = LC_50_ of the most toxic compound × 100/LC_50_ of the tested compound.

**Table 13:** Lethal time (LT) values (per day) and time potency of nanoemulsions of *Rheum rhabarbarum* and *Aloe vera* post-treatment of *Hyalomma dromedarii* males.

Plant extracts	Concentrations								
	15%			8%			3.5%		
	LT_50_ (Lower–upper)	LT_90_ (Lower–upper)	Time Potency	LT_50_ (Lower–upper)	LT_90_ (Lower–upper)	Time potency	LT_50_ (Lower–upper)	LT_90_ (Lower–upper)	Time potency
*Aloe vera*	0.898 (0.415–1.34)	4.79 (3.54–7.73)	1.0	0.931 (0.27–1.58)	11.3 (9.07–34.24)	1.0	1.75 (0.79–2.61)	22.81 (12.16–103.70**)**	1.0
*Rheum rhabarbarum*	0.839 (0.381–1.25)	4.08 (3.04–6.37)	1.07	0.821 (0.21–1.43)	9.77 (6.13–27.79)	1.1	1.34 (0.45–2.16)	20.97 (11.0–110.69)	1.3
Reference (lowest toxicity)	Aloe vera								

	Time potency of before and after nanoemulsions of *Rheum rhabarbarum* and *Aloe vera*								
	15%		3.5%						
	LT_50_	LT_90_	LT_50_	LT_90_					
*Aloe vera*	2.1	4.1	3.5	3.3					
*Rheum rhabarbarum*	2.0	3.1	3.8	2.8					
Reference	*Extracts before nanoformulations*								

Times potency = LT_50_ of the least toxic plant extract/LT_50_ of each tested plant extract.

The finding of this study came along with a previous study that used different concentrations of methanol leaf extract, green synthesized silver (AgNPs), and chitosan nanoparticles (CsNPs) using *A. vera* and *Nerium oleander* against *Musca domestica* and indicated that the nanoparticles were more potent than the methanol extract^97^. Furthermore, a corresponding study indicated that the larvicidal effect of silver nanoparticles AgNPs and CsNPs encapsulated *A. vera* gel extract against *M. domestica* was documented and their relative efficacies were almost 40.65 and 148.51 times more effective than the *A. vera* crude extrac^82^.

A similar study revealed the acaricidal effect of the aqueous extracts of *C. molmol* and *Z. officinale* against *H. dromedarii* in Egypt as their MO% reached 96 and 84.01%, respectively, 15 days PT with 17%, whereas complete MO was reached seven and nine days PT with 12% of their corresponding AgNPs extracts, synthesized physically via laser ablation. Their LC_50_ values reached 10.37, 12.81, 2.38, and 4.12%, respectively, three days PT and their LT_50_ values were 5.6, 6.73, 2.25, and 3.56 days, respectively, PT with 4%. Such extracts reduced *R. (Boophilus) microplu*s three days PT of naturally infested cattle by 54.45, 45.73, 100, and 100%, respectively, whereas such ticks acquired resistance against Deltamethrin (Butox®)^10^. In addition, AgNPs of *R. rhabarbarum* have antibacterial activity towards Gram-positive (+ve) strains of *Staphylococcus aureus* and Gram-negative (−ve) strains of *Escherichia coli*^98^.

### Insect growth regulating effects of nanoemulsions against *H*.* dromedarii*

This study illustrated the significant (*p* < 0.05) adverse effect of nanoemulsions of *A. vera* and *R. rhabarbarum* as IGRs on the reproduction of the engorged females. After treatment with their lowest concentrations (0.75%), the hatchability%, the number of hatched eggs, and the weights of engorged females and egg masses were 61.9%, 116.66, 51.33, and 2.86 g as well as 51.9%, 138.33, 51.0, and 3.93 gm, respectively. Whereas PT with the highest concentration, 25%, egg production was suppressed and engorged female weights were 48.50 and 51.50g, respectively. Nanoemulsions of both extracts adversely affected the reproductive potential of treated engorged females than those of the crude ethanol extracts (Table 14). A similar study proved the IGR effect of AgNPs and CsNPs encapsulated *A. vera* gel extract after treatment of larvae of *M. domestica* as they prolonged larval duration and reduced the pupation and adult emergence rates^82^.

**Table 14:** The insect growth regulating effect of nanoemulsion extracts against *Hyalomma dromedarii* engorged females.

	Conc. %	Number of Hatched eggs (Mean ± SD)	hatchability%	Engorged female weight (gm) (Mean ± SD)	Eggs weight(gm) (Mean ± SD)
*Aloe vera*	Control	1203.33 ± 323.04^a^	97.3	77.33 ± 6.83^a^	38.43 ± 9.10^a^
	0.75	116.66 ± 54.65^b^	61.9	51.33 ± 2.58^b^	2.86 ± 0.79^b^
	1.5	71.66 ± 47.08^b^	50.6	52.16 ± 2.92^b^	0.81 ± 1.27^b^
	3	7.5 ± 11.72^b^	30.0	51.66 ± 3.20^b^	0.60 ± 1.36^b^
	12	0.00 ± 0.00^b^	0.0	54.0 ± 3.08^b^	0.00 ± 0.0^b^
	25	0.00 ± 0.00^b^	0.0	48.50 ± 11.30^b^	0.00 ± 0.0^b^
*Rheum rhabarbarum*	Control	1203.33 ± 323.04^a^	97.3	77.33 ± 6.83^a^	38.43 ± 9.10
	0.75	138.33 ± 87.27^b^	51.9	51.00 ± 3.09^b^	3.93 ± 2.11^a^
	1.5	79.16 ± 78.12^b^	44.2	51.00 ± 3.09^b^	0.44 ± 00.31^b^
	3	10.83 ± 17.72^b^	17.6	50.66 ± 2.16^b^	0.34 ± 0.76^b^
	12	0.33 ± 0.81^b^	4.3	49.33 ± 10.15^b^	0.13 ± 0.32^b^
	25	0.00 ± 0.00^b^	0.0	51.50 ± 3.14^b^	0.00 ± 0.0^b^

Means followed by the same letter in the same column were not significantly different by ANOVA (*p* > 0:05).

### Biochemical characterization

Enzymes are usually used as reliable indicators for evaluating the impact of the applied toxic materials against insects^99,100^; the mechanisms of insecticide resistance mostly include enzymes involved in the detoxification of carbamates, organophosphates, pyrethroids, and growth regulators such as non-specific esterase, Glutathione-S-transferase (GSTs), and P450-mediated monooxygenase (MFOs)^100^. Generally*,* biochemical analyses of the present work revealed that the total protein, carbohydrates, AChE, Alpha esterase, and Amylase were affected after treatments; their values PT with nanoformulations were significantly (*p* < 0.05) more affected than those of the control group, except for the total protein level. The level of Alpha esterase protein was significantly (*p* < 0.05) increased in the case of *Rheum* extract (Table 15).

**Table 15:** Biochemical parameters after treatment of male *Hyalomma dromedarii* with plant extract.

Biochemical tests	Treated male *Hyalomma dromedarii* Mean ± SD				Control mean ± SD
	*Aloe vera*	*Rheum rhabarbarum*	*Aloe vera* nanoemulsion	*Rheum rhabarbarum* nanoemulsion	
Total protein (mg/L)	56.7 ± 1.5^c^	68.1 ± 2.7^b^	73.13 ± 1^a,b^	77.13 ± 1.7^a^	76.9 ± 3^a^
Total carbohydrates (mg/L)	8.6 ± 0.6^a^	4.4 ± 0.4^b^	2.4 ± 0.2^c^	2.6 ± 0.3^c^	8.9 ± 0.9^a^
Acetylcholinesterase (AChE) (mg AchBr/min/mg protein)	102.3 ± 4.9^a^	93.0 ± 3^a,b^	85.7 ± 4.7^b,c^	81.0 ± 2^c^	94.5 ± 5^a,b^
Alpha esterase (mg α-naphthol/min/mg protein)	65.3 ± 5^c^	136.0 ± 10.7^a^	53.7 ± 1.5^c^	89.7 ± 4.4^b^	59.0 ± 3.6^c^
Amylase (mg glucose/ min/ mg protein)	10.1 ± 0.9^a^	6.6 ± 0.6^b^	4.4 ± 0.2^c^	3.2 ± 0.2^c^	7.9 ± 0.8^b^

Means followed by the same letter on the same raw were not significantly different by ANOVA (*p* > 0:05).

Analog results revealed that *Citrus limetta* seed oil disturbed the defensive and target enzymes of *Rhipicephalus microplus* via reducing the levels of SOD, GST, MAO, and AChE and increasing NOS level in ticks when compared with the control group^95^. Furthermore, azadirachtin significantly inhibited the activity of AChE when compared with control of *N*. *lugens*^96^. Essential oils can diminish esterase, glutathione S-transferases (GSTs) activities, and the total carbohydrate, lipid, and protein contents in *Tribolium castaneum*^101,102^.

*Aloe* and *Rheum* extracts in this study significantly decreased the total protein as compared to the control group. Similarly, reduction in total protein content (representing defensive and target enzymes) is a common occurrence in insects after treatment with toxic compounds, but increased after treatment of a field strain of the mosquito, *Culex pipiens*, with some IGRs like lufenuron and novaluron^100^. Such variation might be due to using different species and compounds.

In this study, there was an increased level of AChE after treatment with *A. vera* ethanol extract but decreased PT with its nanoemulsion and *R. rhabarbarum* extracts. Similarly, essential oils as well as carvacrol have an AChE inhibitory effect indicating that the position of the hydroxyl group in the structure plays an important role^85^. AChE is the main physiological target of many synthetic acaricides such as organophosphate (OP) and carbamate leading to overstimulation of the neurons and rapid twitching of the muscles, convulsions, and death. Treatment with *A. vera* extract significantly increased AChE level in this work. A parallel study indicated that imidacloprid significantly increased AChE, Glutathione-S-transferases, GSTs, and activities per protein content of the water flea *Daphnia magna* (Daphniidae: Anomopoda) 21 days PT^103^. A similar recent study showed elevated levels of AChE, α and β esterases, and GSH after treatment of *Cx. pipiens* with IGRs like lufenuron^100^.

In contrast, AChE level was decreased PT with *Rheum* nanoemulsion in this study. Alike, AchE activity was significantly inhibited in larvae of *R. annulatus* PT with fennel oil and its main constituents, trans-anethole and fenchone^104^; and larvae of *Rhipicephalus microplus* PT with the *n*-hexane extract of *Calea serrate*^105^^.^*.* Moreover, emodin, extracted from *Rheum palmatum,* exhibited significant AChE and GST inhibitory activities when applied against *Nilarparvata lugens* and *Mythimna separate* (IC_50_ = of 11.36 and 4.18 μg/mL, respectively)^93^.

It is worth mentioning that silver and graphene oxide nanoparticles affected insect antioxidant and detoxifying enzymes, inducing oxidative stress and cellular death^106^. The pesticidal action of the synthesized green nanoparticles could be induced because of their penetration into insect exoskeleton, ability to bind to the sulfur element with proteins or DNA phosphorylation, and rapid denaturation of saturation^106,107^.

Plant extracts contain secondary compounds derived from plants that perform useful functions against insects by acting as repellents, antifeedants, and toxins and have provided alternative resources for insect control. They are also characterized by their biodegradation and minimal harmful effects on non-target organisms^19,55,56^**.** In general, botanicals could be recognized as safe^19–21^. *R. rhabarbarum* is a medicinal edible plant consumed worldwide^59^. *A. vera* has anticoccidial, antibacterial, and immunomodulatory effects; therefore, it enhances the intestinal health and performance of birds when used as a safe feed additive^108^. The aqueous and hydroethanolic extracts of *Tabernaemontana elegans; Calpurnia aurea, Schkuhria pinnata,* and *Aloe rupestri*s (leaves, stems, whole plant, and leaves, respectively) not only effectively controlled *R. turanicus*, but also were safe or very safe on human Vero kidney and liver HepG2 cells^89^. Temulawak (*Curcuma xanthorrhiza* Roxb) nanoemulsion is safe and improves chickens’ productivity and performance and could prevent the risks of antibiotic residues and resistance^109^. No symptoms of skin irritation or abnormal health observation were observed among operators as well as birds^44^ and buffaloes^36^, post spraying and pour-on applications, respectively, of essential oils; rabbits PT with aqueous neem extract^14^; and cattle after spot-on application with aqueous and silver nanoformulations of *C. molmol* and *Z. officinale*^10^.

## Conclusions

This investigation proved that the novel ethanol extracts of *A. vera* and *R. rhabarbarum* and their nanoemulsion induced effective acaricidal and growth-regulating effects against *H. dromedarii*. Nanoformulations of *A. vera* and *R. rhabarbarum* enhanced the efficacy of the ethanol extracts 1.5- 2.5 times three days PT, and 1-7 times five days PT and accelerated the speed of killing ticks 2-4 times faster than the ethanol extracts. They also adversely affected the reproductive potential of engorged females. Consequently, they could prevent tick bites and their associated diseases as eco-friendly acaricides. It is indicated from this investigation that botanicals could be used for the progress of benign and eco-friendly acaricides against *H. dromedarii*. Further studies could be directed towards in vivo and ecotoxicological studies of *A. vera* and *R. rhabarbarum*. 

## Data Availability

The datasets used and/or analyzed during the current study are available from the corresponding author on reasonable request**.**
